# Depression and Anxiety of Portuguese University Students: A Cross-Sectional Study about Prevalence and Associated Factors

**DOI:** 10.1155/2024/5528350

**Published:** 2024-01-12

**Authors:** Pedro Amaro, César Fonseca, Anabela Afonso, Gonçalo Jacinto, Luís Gomes, Hélder Pereira, Helena José, Celso Silva, Andreia Lima, Helena Arco, João Nabais, Manuel Lopes, Anabela Pereira, Isabel Fragoeiro, Lara Guedes Pinho

**Affiliations:** ^1^Comprehensive Health Research Centre (CHRC), Universidade de Évora, 7002-554 Évora, Portugal; ^2^Institute for Advanced Studies and Research, Universidade de Évora, 7002-554 Évora, Portugal; ^3^VALORIZA-Research Centre for Endogenous Resource Valorization-Polytechnic Institute of Portalegre, 7300-555 Portalegre, Portugal; ^4^Nursing Department, Universidade de Évora, 7000-811 Évora, Portugal; ^5^CIMA, IIFA, Universidade de Évora, 7002-554 Évora, Portugal; ^6^Mathematics Department, ECT, Universidade de Évora, 7000-671 Évora, Portugal; ^7^School of Health, University of the Azores, 9500-321 Ponta Delgada, Portugal; ^8^NURSE'IN UIESI-Nurse Research Unit for South and Islands, Polytechnic Institute of Setúbal, 2910-761 Setúbal, Portugal; ^9^Escola Superior de Saúde Atlântica, 2730-036, Barcarena, Portugal; ^10^Health Sciences Research Unit: Nursing, 3046-851 Coimbra, Portugal; ^11^School of Health, Instituto Politécnico de Beja, 7800-295 Beja, Portugal; ^12^CINTESIS@RISE, Institute of Research, Innovation and Development, University of Porto, 4200-450 Porto, Portugal; ^13^Higher School of Health Fernando Pessoa, 4200-253 Porto, Portugal; ^14^Department of Health Sciences and Technologies, Polytechnic Institute of Portalegre, 7300-555 Portalegre, Portugal; ^15^Departamento de Ciências Médicas e da Saúde, Escola de Saúde e Desenvolvimento Humano, Universidade de Évora, 7000-671 Évora, Portugal; ^16^Centre for Research in Education and Psychology (CIEP-EU), 4200-135 Porto, Portugal; ^17^Portugal Williams James Center for Research (WJCR), 1149-041 Lisboa, Portugal; ^18^Department of Psychology, Universidade de Évora, 7000 Évora, Portugal; ^19^School of Health, University of Madeira, 9000-082 Funchal, Portugal

## Abstract

**Background:**

The mental health of university students has worsened over time, and it is young people who have suffered the most from the COVID-19 pandemic in terms of mental health. Anxiety and depression are the most common symptoms reported by university students and are often the cause of disabilities, either in academic performance or in other spheres of life.

**Aim:**

The aim of this study was to both assess the prevalence of depressive and anxiety symptoms in Portuguese university students and analyze the factors associated with these symptoms.

**Methods:**

A quantitative cross-sectional study with a sample of 3,399 university students from seven Portuguese higher education institutions was conducted. The following questionnaires were used: the generalized anxiety disorder assessment scale (GAD-7), the patient health questionnaire (PHQ-9), and a study-created sociodemographic questionnaire. The Kendall correlation coefficient, chi-square test of independence, Spearman correlation coefficient, Shapiro-Wilk test, Mann–Whitney-Wilcoxon test, and Kruskall-Wallis test were used to analyze the association between variables. The statistical analysis was done using the software R Statistics (Version 4.0.4), using a significance level of 0.05.

**Results:**

Mild to severe anxiety symptoms were reported by 75% of the participants, and 61.2% described mild to severe depressive symptoms. Of the sample, 19.5% reported a previous diagnosis of a mental disorder, with 38.7% diagnosed after the pandemic began. Additionally, 23% reported taking medication for mental health issues, and 26.7% had considered self-harm or harbored thoughts of being “better off dead.” The study found lower anxiety and depressive symptoms (*p* < 0.05) among men, students with higher socioeconomic status, those who frequently traveled home, individuals without prior mental health diagnoses, those with better academic performance, and those who avoided substances like coffee, tobacco, cannabis, and other illegal psychoactive substances. Interestingly, students in romantic relationships exhibited more anxiety symptoms (*p* < 0.05). Moreover, participants who believed they had experienced moral or sexual harassment displayed higher levels of anxiety and depressive symptoms (*p* < 0.001).

**Conclusions:**

There was a decrease in the mental health of university students after the pandemic compared to prepandemic studies, and the proportion of students with anxiety and depressive symptoms was alarming. There is an urgent need to implement programs in universities to promote students' mental health.

## 1. Introduction

Getting into a university is a complex transition that involves several changes. It requires students to have a broad set of skills to be able to deal positively with all the demands they face. At this stage of life, university students create new emotional bonds; doubts about their chosen career may arise; greater independence in problem-solving is required; there is an increased level of demand for knowledge; and there is the acquisition of more complex responsibilities [1]. In this sense, difficulties may arise at various levels: organizational (e.g., too many tasks to perform and lack of knowledge about teaching and assessment methods); interpersonal or social (e.g., new relationship patterns with teachers and peers and the need for new social support networks); and personal (e.g., lack of family support and socioeconomic difficulties) [2]. These difficulties tend to lead to high levels or permanent states of stress that impair the well-being, adaptation, and academic engagement of university students [3]. Also, studies show that 75% of all mental disorders have their onset before the age of 24 years, thus the increased vulnerability of university students [4].

In 2020, there was a pandemic around the world that led to changes in the population's health. Studies indicate that the age group that suffered the most mental disorders from the pandemic was the youngest [5, 6]. The measures taken due to the COVID-19 pandemic focused on the prevention of physical illnesses and negatively affected university students socially, physically, and mentally [7–9]. The context experienced during COVID-19 potentiated the development or increase of mental health disorders, such as anxiety and depression [10]. In Portugal, all academic celebrations and nonteaching or scientific activities were forbidden in higher education institutions [11]. At the end of March 2020, all educational establishments were closed, and online emergency learning started. All activities that required the physical presence of students, such as hospital internships, were interrupted and postponed. This context required a readaptation of both university students and the education system worldwide, with an impact on mental health [12].

Studies have shown a worrying prevalence of mental health problems among university students in the prepandemic phase, with high levels of depression and anxiety [13–16]. In Portugal, data from the 2017–2018 Polytechnic Higher Education Students' Health Behaviors and Well-Being report show that 16.6% had a low level of mental health, and 10% reported self-injurious behavior. Mental health had a positive association with high socioeconomic status, stable affective relationships, academic performance, and better sleep habits [17]. A systematic review concluded that in the pandemic context, there was a considerable increase in mental distress in university students compared to the prepandemic phase, with risk factors such as having friends or family members infected with COVID-19 or challenges imposed by online emergency learning [18]. The results of a meta-analysis showed that the prevalence of depression (39%) or anxiety (36%) among university students increased greatly during COVID-19, and that the mental health of populations differed between countries [19].

However, psychological distress among university students has been on the rise since 2010 [20]. Studies indicate some factors that can influence students' mental health, such as being displaced from one's family residence, academic difficulties/low performance, a previous mental disorder [21], adverse factors in childhood or recent stressors [22], or poor sleep quality [23, 24]. Studies have also shown that the area of training (i.e., a student's major) influenced reported levels of mental distress (e.g., anxiety and depression), where the prevalence rate of anxiety in medical students was substantially higher than in the general population [25, 26] or nonmedical students [27]. Also, moral and sexual harassment coupled with deteriorating social relationships characterized by greater rigidity and verbal and psychological violence (such as bullying, competitiveness, productivity, and demanding standards) can lead to greater mental distress [28].

These vulnerability factors can negatively influence the academic path, making students a high-risk group for the development of anxiety and depression, which can conduce low academic performance, decreased quality of life, and dropout [1, 29, 30]. Studies indicate that entering a university can cause psychological distress, even for those without preexisting mental health problems [31, 32]. Thus, the mental health of university students has become a growing public health concern [33].

Of the mentioned reviews, there is a lack of studies conducted in Portugal, and most of the studies were conducted in countries outside Europe. Therefore, it is important to understand whether the results are similar or different in Portugal to plan interventional measures. This study contributes to the early identification of anxiety and depression in Portuguese university students (through the association with associated factors) and provides a basis for the creation of mental health promotion programs for them. It might also contribute to university management decisions based on the current state of students' mental health.

Given the above, this study is aimed at assessing the prevalence of depressive and anxiety symptoms in Portuguese university students and analyzing the factors associated with these symptoms.

To answer these questions, the following research questions were formulated:

1. What is the prevalence of depressive and anxiety symptoms in Portuguese university students?
2. What factors are associated with depressive and anxiety symptoms in Portuguese university students?

## 2. Materials and Methods

### 2.1. Study Design

This was a quantitative cross-sectional study, using the STROBE guidelines [34]. It was registered with the Open Science Framework (OSF): doi:10.17605/OSF.IO/WJ7TD.

### 2.2. Population and Participant Selection

The target population for this study was university students attending Portuguese higher education institutions.

The inclusion criterion for the sample was (1) being a university student at one of the study's seven partner institutions and (2) fluency in Portuguese (including international students). These students had to have access to the Internet and were chosen using a nonprobabilistic convenience sampling method.

### 2.3. Participants

A total of 3,653 responses were obtained, but 254 were excluded because we did not receive answers to all the GAD-7 and PHQ-9 items. The final sample of this study consisted of 3,399 university students from seven Portuguese higher education institutions (Universidade de Évora, University of the Azores, University of Madeira, Escola Superior de Saúde Atlântica, Polytechnic Institute of Beja, Polytechnic Institute of Portalegre, and University Fernando Pessoa), i.e., the overall response rate was 12.5%. There was no monetary compensation for participants.

### 2.4. Instruments

The sociodemographic questionnaire contained questions assessing age, sex, emotional relationship, sexual orientation, nationality, subjective socioeconomic level, frequency of going to the official residence (i.e., home), and who students lived with during school.

The data on attending higher education included study major/scientific area (using the National Classification of Education and Training Areas); cycle of study; year of the course attended; perception of academic performance (How do you rate your academic performance?); type of education (public; private); and student-worker status, with quantification of the number of hours worked per week.

The following mental health data were also collected: perception of how COVID-19 affected their mental health (How did the COVID-19 pandemic, between 2020 and 2022, affect your mental health?); previous diagnosis of mental disorders (whether the mental disorder was diagnosed before or during COVID-19); whether the student had psychiatric consultations; and whether they took medication for mental health problems, what type of medication they took, and whether it was prescribed by a doctor.

Regarding moral and sexual harassment, the concept of moral harassment was defined as “any abusive conduct of a psychological nature, frequent and intentional, through attitudes, gestures, words, or writing that may harm physical or psychological integrity, generating a feeling of exclusion from the environment and from social coexistence” [35], and the concept of sexual harassment was defined as “any behavior, or disclosure, by words, or actions, of a sexual nature, not intended by the person to whom it is directed, and which proves offensive” [36]. The participants were asked whether they experienced these, the place where the harassment was experienced (at the university; outside of the university), and the type of aggressor (teachers, student colleagues, work colleagues, nonteaching staff at the university, romantic partner(s), relatives, etc.).

Participants also reported on their drinking and drug intake (coffee or other energy drinks; cigarettes; alcohol; cannabis; and other psychoactive substances such as cocaine or ecstasy), the frequency of consumption, and in the case of alcohol, the quantity used.

The generalized anxiety disorder assessment scale (GAD-7), Portuguese version, had its psychometric properties tested by Sousa et al. [34]. The GAD-7 [37] is a self-administered questionnaire used as both a screening tool and a measure of severity for people with generalized anxiety disorders. It consists of seven items: (1) feeling nervous, anxious, or on edge; (2) not being able to stop or control worry; (3) worrying too much about different things; (4) difficulty relaxing; (5) being so restless that it is difficult to sit still; (6) being easily bored or irritable; and (7) feeling afraid as if something terrible might happen. The period of measurement is the previous 2 weeks, and using a 4-point Likert scale from “not at all” to “almost every day,” the person is asked how often they have been bothered by any of the problems presented. A score < 5 was defined as no symptomatology, from 5 to 9 as mild, scores from 10 to 14 as moderate, and scores ≥ 15 as severe. Higher values indicate a greater presence of anxiety symptomatology. The GAD-7 has correlated highly not only with specific measures of anxiety but also with measures of disability, presenting excellent psychometric properties, high discriminant capacity, brevity, and rapid administration. Cronbach's alpha of the GAD-7 in our sample was 0.91 (excellent internal consistency).

The patient health questionnaire (PHQ-9), Portuguese version, had its psychometric properties tested by Monteiro et al. [38]. The Portuguese version of the PHQ-9 has a satisfactory internal consistency, Cronbach's alpha of 0.86 [39]. The PHQ-9 [38] is a self-report measure consisting of nine items to assess depressive symptomatology. Participants are asked to report symptoms experienced during the 2 weeks before completing the questionnaire, using a 4-point scale. Scores for each item on the PHQ-9 range from 0 (never), 1 (several days), 2 (more than half of the days), to 3 (almost every day). Summed scores range from 0 to 27, with higher scores being associated with worse depression. As a category of depression severity, scores indicate minimal (0–4), mild (5–9), moderate (10–14), moderately severe (15–19), or severe (20–27) depression. Cronbach's alpha of the PHQ-9 in our sample was 0.90 (excellent internal consistency).

### 2.5. Procedures

Data collection began on October 10, 2022 (International Mental Health Day), and ended in December 2022. The online questionnaire was constructed using Google Forms and was sent by email to all students at the universities involved. The students were also asked to complete the questionnaire in their classrooms. The average time to complete the questionnaire was around 15 minutes.

### 2.6. Ethical Aspects

The present study was conducted in accordance with the Declaration of Helsinki, with its modifications or similar ethical principles [40]. Participants were informed about the scope and objectives of the study, as well as the benefits and confidentiality conditions. Only after giving informed consent by clicking “yes” was it possible to start the questionnaire, with participants knowing they could leave the study at any time. The Ethics Committee of the University of Évora preapproved the study (Document no. 22067, dated September 24, 2022). The Data Protection Officer of the University of Évora guarantees that the processing of personal data complies with the legislation in force.

### 2.7. Statistical Analysis

Initially, an analysis of nonresponses was conducted. Since no patterns were identified in these nonresponses, and the nonresponse rate was low (mean = 0.63% ± 0.64%, range = 0.1%–2.5%), it was assumed that the nonresponses were completely random. Therefore, we will report the valid proportions.

The Kendall correlation coefficient was used to measure the correlation between the frequency of consumption of some products and the items of the scales (GAD-7, PHQ-9).

The chi-square test of independence was used to determine whether statistically significant relationships existed between the self-assessment of academic performance and study cycle, as well as between the existence of financial support and the type of university, and between financial support and the study cycle.

The Spearman correlation coefficient was used to measure the correlation between the score scales (GAD-7, PHQ-9) and age.

The Shapiro-Wilk test, as well as the analysis of the skewness and kurtosis coefficients, was used to evaluate the normality assumption of the numeric variables. The homogeneity of variances was evaluated with the Levene test.

Due to the strong violation of the normality assumption, the Mann–Whitney-Wilcoxon test was used to compare the scale scores between two groups (for instance, sex, love relationship, and displacement from home).

The Kruskall-Wallis test was used to compare the scale scores in more than two groups (socioeconomic level, frequency of going home, and academic performance), due to the strong violation of the normality assumption. Under the rejection of the null hypothesis, the Dunn test was used, or when the assumption of equal variance was held, the Games-Howell test was used.

The statistical analysis was done using the software R Statistics (version 4.0.4; the R Foundation, Vienna, Austria). A significance level of 0.05 was used.

## 3. Results

### 3.1. Sociodemographic and Clinical Data

The sample's mean age was 21.61 (±5.91); participants were between 17 and 69 years old, with 80.5% younger than 23, and 7.2% older than 30. Most of the participants were female (68%). The other sociodemographic characteristics and academic characteristics can be seen in Table 1.

### 3.2. Mental Health Data

**Figure 1:**
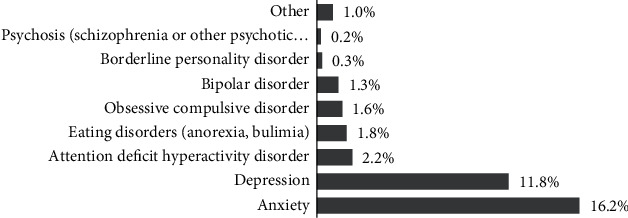
Mental disorder diagnoses reported by students (*n* = 3,399).

Regarding the clinical data, 19.5% of the participants reported having a diagnosed mental disorder, and of these, 38.7% reported that the diagnosis was made after the beginning of the COVID-19 pandemic. The most mentioned diagnoses were anxiety and depression, with 10.2% of the students reporting both diagnoses (Figure 1). Only 23.6% of the participants reported ever having had psychiatric consultations, and 23.0% reported taking medication for some mental problem. Of students who took medication, 27.7% reported that the medication was not prescribed by a physician. However, only 10.9% were natural medicines that did not require a prescription. Students reported taking these types of medications: natural (10.9%), antidepressants (8.5%), benzodiazepines (6.7%), antipsychotics (1.6%), and mood stabilizers (1.1%).

**Figure 2:**
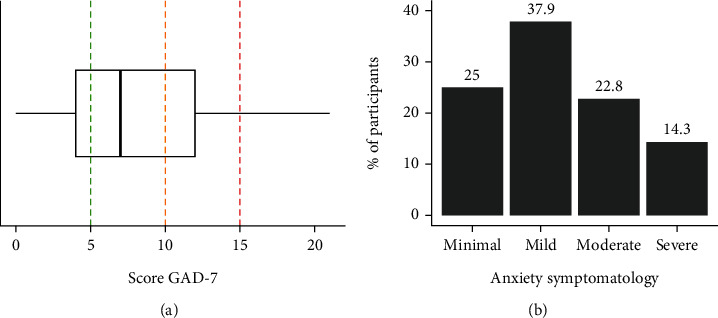
Boxplot for the score GAD-7 (a) and % of anxiety symptomatology (b).

Regarding anxiety symptoms, participants scored between 0 and 21 on the GAD-7 (total scale); the mean was 8.42 ± 5.40, and 37.1% had moderate or severe symptoms (Figure 2). During the prior 14 days before survey completion, the problems that most affected the participants for several days were “nervous, anxiety, or irritable” (48.2%), “I worried too much about different issues” (44.6%), “I had trouble relaxing” (43.8%), and “I was easily annoyed or irritable” (40.3%). The problems (“I felt afraid, as if something awful might happen” (38.1%) and “I was so restless that it was hard to sit still” (37.2%)) affected the participants the least. It should be noted that each of the problems mentioned in this scale was experienced almost every day by 8–17% of the participants, most notably “worrying too much about different issues” (16.9%).

**Figure 3:**
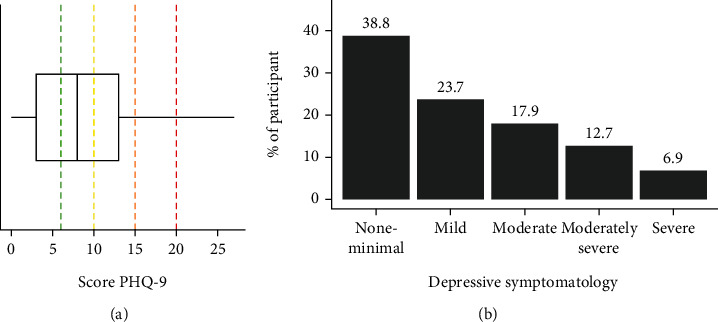
Boxplot for the score PHQ-9 (a) and % of depressive symptomatology (b).

Regarding depressive symptoms, participants scored between 0 and 27 on the PHQ-9 (total scale); the mean was 8.59 ± 6.43, and 38.8% of the participants had moderate or several symptoms (Figure 3). Over the prior 2 weeks to survey completion, the problems that bothered participants most often (at least more than half of the days) were “feeling tired or having little energy” (41.2%) and “trouble falling or staying asleep or sleeping too much” (37.6%). “Thoughts that you would be better off dead or of hurting yourself in some way” was reported by 26.7%, and 14.9% reported “moving or speaking so slowly that other people could have noticed, or the opposite—being so fidgety or restless that you have been moving around a lot more than usual.”

It was reported by 31% of the participants that the problems referred to above caused extreme difficulty in their lives, 45.5% reported little difficulty, and 23.5% reported “not difficult/not applicable.”

### 3.3. Substance Use

Alcohol was the substance most consumed by students (84.7%), followed by coffee (80.6%), tobacco (68.7%), cannabis (14.1%), and other psychoactive substances (2.4%) such as cocaine and ecstasy. Of the participants, 63.8% consumed four to five servings of alcohol on the same occasion.

### 3.4. Moral and Sexual Harassment

Regarding the concept of moral harassment presented in the questionnaire, 50.2% of students considered that they had suffered from moral harassment; 191 (5.6%) participants did not answer this question, with 187 (5.5%) stating they preferred not to answer. The aggression almost always occurred outside the university (93%), with 10% of the participants (*n* = 188) who were morally assaulted not identifying any aggressor. The aggressors reported were family members (26.7%), student colleagues at the university (25.1%), coworkers (22.3%), a love partner (14.9%), or professors (11.7%). In the analysis of the other aggressors mentioned, classmates/schoolmates (*n* = 168; 10%) and friends (*n* = 65; 4%) stood out.

Considering the concept of sexual harassment presented in the questionnaire, 34.8% of the participants reported they have experienced sexual harassment, while 126 (3.7%) participants did not answer this question, and 119 (3.5%) said they preferred not to answer. Sexual assault almost always occurred outside the university (97.5%). A total of 20% of the sexually assaulted participants (*n* = 233) did not identify an aggressor, and the most identified aggressors were nonfaculty university staff (14%) or a love partner (10.5%), followed by coworkers (8.1%), student colleagues at the university (7.3%), relatives (6.4%), or professors (2.9%). In the analysis of the other aggressors mentioned, there was a strong emphasis on strangers (*n* = 383; 33%).

### 3.5. Association among Variables

There was no significant correlation between age and anxiety (*r* = −0.008; *p* = 0.650) or depression (*r* = −0.024; *p* = 0.156) scores.

Significant differences in the levels of anxiety and depression were found in some of the sociodemographic and academic characteristics and for some of the substance consumption variables (Table 2).

A significant association (*p* < 0.001) was found between depressive and anxiety symptomatology and the area of study. Students from arts/humanities majors were more likely to report higher anxiety and depressive symptomatology than students studying health and engineering, manufacturing, or construction.

**Figure 4:**
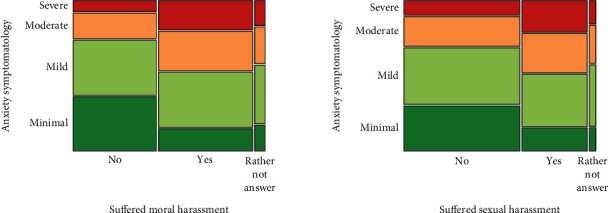
Relation between anxiety symptomatology and the type of harassment suffered.

**Figure 5:**
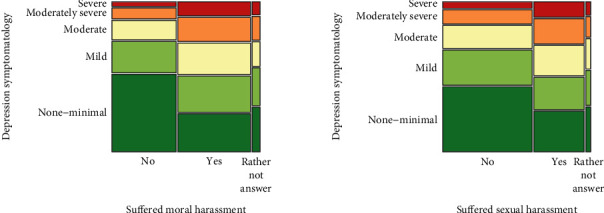
Relation between depression symptomatology and the type of harassment suffered.

A significant association was observed between anxiety symptomatology and a student suffering moral (*p* < 0.001) or sexual (*p* < 0.001) harassment and between depressive symptomatology and these two kinds of harassment (both *p* < 0.001) (Figures 4 and 5). A significant association was also observed between sex and moral or sexual harassment (*p* < 0.001), where women reported having suffered more than men from moral and sexual harassment. Students who suffered moral or sexual harassment were more likely than others to report at least moderate anxiety symptoms, as well as at least moderately severe depressive symptoms.

## 4. Discussion

### 4.1. What Is the Prevalence of Depression and Anxiety Symptoms in Portuguese University Students?

Our results showed higher levels of depressive and anxiety symptoms in Portuguese university students when compared with the results of studies carried out on Portuguese higher education students in prepandemic periods. We found a higher prevalence of anxiety symptoms in our study compared to a study conducted in Portugal before COVID-19 that used the GAD-7 [41]. In that study, 60.2% of university students presented anxiety symptoms, and in our study, the percentage was higher (75%). Another study conducted in Portugal during the pandemic (April 2020 to October 2020) found that 36.2% of university students had anxiety symptoms (mild to extremely severe on the DASS-21) [42]. Another Portuguese survey conducted in private universities 1 year later (June 15 to October 15, 2021) showed that 35.9% of the students had anxiety symptoms (mild to severe on the DASS-21) [43]. In our study, the numbers were higher, including 75% reporting mild to severe anxiety symptoms on the GAD-7. Regarding depressive symptoms, the results are similar. A study conducted in prepandemic Portugal with university students concluded that they had lower levels of depression (30.20%) [17]. In the same Portuguese studies above, it was found that 28.5% (2020 data collection) [42] and 37.5% (2021 data collection) [43] of students had depressive symptoms, with the percentage being higher in our study (61.2%; 2022 data collection). For other countries, we found similar results. Although it is necessary to analyze the data with caution since different scales were used, we found that anxiety and depressive symptoms were much higher when compared to the Portuguese results from the 2020 and 2021 pandemic years. Reviewing a meta-analysis with studies from 15 countries involving 706,415 college students, it was found that the prevalence of depressive symptoms increased from 21% (95% CI: 16–25%) before March 2020 to 54% (95% CI: 40–67%) after March 2020 [19]. Similarly, anxiety symptoms increased from 19% (95% CI: 13–25%) to 37% (95% CI: 26–48%) [19]. A systematic literature review involving medical students in Asia, the Middle East, South America, North America, Europe, Australia, and Africa showed that COVID-19 had a negative impact globally on the mental health of university students, with increased levels of depression and anxiety related to the transition to online education, social isolation, reduced social support, exposure to high-risk environments, disruption of clinical teaching, or decreased self-efficacy to cope with the new living contexts [44]. These international studies, along with studies carried out with Portuguese students, reinforce the fact that the COVID-19 pandemic negatively affected students' mental health (depression and anxiety).

It is important to point out that 26.7% of the students reported that they had thoughts about being “better off dead” or getting hurt in some way. This percentage is higher than that of a study conducted with Portuguese university students before the pandemic, which found that 7.8% had severe suicidal ideation—the phenomenon being complex, of multifactorial etiology, and related to sociodemographic factors [45]. Another study with Saudi students showed that 32% had thoughts that they were better off dead or hurting themselves in some way [46]. This is an issue implying that universities should be creating mechanisms to assess suicidal ideation and intervene early, preventing suicide. A systematic review of the literature highlighted the gap that exists in the development of support programs for university students with self-harm behaviors [47].

Consistent with previous studies, the present study also revealed an association between previous mental disorders and poorer mental health during higher education [21, 48]. This factor may be related to the fact that there is already an existing vulnerability at a stage prior to entering higher education; thus, there is a need for constant and objective monitoring of university students.

### 4.2. What Factors Are Associated with Depression and Anxiety Symptoms in Portuguese University Students?

We found an association between these symptoms and sex, socioeconomic status, frequency of going home, academic performance, area of study, substance abuse, previous mental disorder, and moral or sexual harassment. Even fewer depressive symptoms were observed in student workers and fewer anxiety symptoms in those who were not in a romantic relationship.

#### 4.2.1. Sociodemographic Characteristics

We found better mental health in male students compared to female students. This is consistent with studies in other countries that found a higher prevalence of depressive and anxiety symptoms in women [20, 49, 50]. A study conducted by Kuehner [51] showed that women are twice as likely to develop depression than men. In contrast, a study conducted in South Africa [52] found no significant difference in depressive symptoms between male and female students. The gender difference in depression or depressive symptoms varies between countries [53]. This is a fact that should be reflected since there are similar results in samples of other age groups. A systematic literature review involving metaregression [54] that assessed the impact of COVID-19 on depressive and anxiety symptoms in the general population in 204 countries and territories showed that, for both disorders, women were more affected than men, with a 27.9% (25.6–30.4) increase in anxiety symptoms compared to a 21.7% (19.3–24.1) increase in men. Regarding depressive symptoms, there was an increase of 29.8% (27.3–32.5) in women and an increase of 24.0% (21.5–26.7) in men.

The present study showed an association between lower socioeconomic status and worse mental health in university students, cohering with prior evidence. A systematic literature review of meta-analyses [55] that included 64 studies (*n* = 100,187) found an association of low socioeconomic status with poorer mental health (OR: 1.60, 95% CI: 1.20–2.14). People with a better socioeconomic status have more access to activities that promote mental health, such as physical exercise, recreational activities, activities that promote socializing (e.g., going out to eat with friends), and better access to health services, including psychiatric or psychological consultations. So, to promote mental health, it is important that the countries' policies involve strategies to improve people's socioeconomic status, providing greater equity in the distribution of wealth and better access to public services, such as education and health.

In the present study, we found data suggesting that loving relationships are related to more anxiety symptomatology, with no significant difference observed per depressive symptoms. Other studies showed that satisfying love relationships are associated with mental well-being that can be affected when the relationship is dysfunctional [56]. More data would be needed to interpret these results since we do not know the type of relationship the anxious students had or whether they lived far from or close to their boyfriend or girlfriend. One finding that may relate to this data is the fact that 10.5% of the students reported experiencing sexual harassment from their love partner, and 14.9% of the participants reported experiencing moral harassment from their love partner. This may be a possible reason for anxiety, but there are other factors that should be investigated.

Among the students displaced from their official residence (i.e., home), the ones who went home frequently (every weekend) reported better mental health than students who went home less frequently. Identical results have been found in other studies, showing that students who are displaced and go homeless frequently have worse mental health [49, 57]. It is also necessary to frame the student's life as a moment where the young adult is still in some ways dependent on their family. One study showed that family functioning can also influence the mental health of university students [58]. It is, therefore, important to prepare students before they enter university in a way that both promotes independent living and maintains regular contact with parents or family members.

The present study showed that student workers reported lower depressive symptoms compared to those who did not work. These data may be related to the fact that student workers may have greater motivation to achieve educational and professional goals, increasing their confidence and emotional resilience. A systematic literature review [59] found that working and studying do not affect the academic performance of university students; however, student workers tend to report a higher rate of depression than students who do not work, which does not accord with our results. Depressive and anxiety symptoms can be associated with difficulty in balancing studies with work and family, lower attendance at tutorial meetings, or difficulty in balancing personal and work life [60].

#### 4.2.2. Academic Data

Another finding from our study was the association between academic performance and depressive and anxiety symptoms, with students with a perceived worse academic performance reporting worse symptoms. This is corroborated by international studies showing that students with poorer mental health had lower academic performance and higher dropout [61, 62]. There is a need to both explore the factors that mediate the relationship between mental disorders and academic performance and develop predictive models of mental disorder [63]. With our cross-sectional study, we cannot infer causality; however, we know that people with depressive symptoms usually have low self-esteem, so the perception of their academic performance will be lower. On the other hand, anxiety and depressive symptoms, such as low motivation or unwillingness, can cause worse academic performance, which influences students' perceptions.

The present study found a significant association between the area of study and depressive and anxiety symptomatology, with students in the arts/humanities reporting higher anxiety and depressive symptomatology. Students of health and engineering, manufacturing, and construction reported relatively less depressive and less anxiety symptomatology. In turn, some studies have found a high prevalence of anxiety and depression in health students [64, 65]. A Portuguese study reported that medical students have more anxiety symptoms (*p* = 0.034) than students in other fields [27]. So, our study's results are relatively different. More research in this area is important since the referenced studies were conducted prepandemic. During the pandemic, it is possible that students of the health sciences found strategies to overcome the difficulties, and those studying the arts did not. Art students had their activities stopped for some time, and this may have affected their mental health uniquely, since these activities are typically mental health promoting.

We did not find a significant difference between first-year students and others. Other studies have found a worrying prevalence of anxiety in first-year students [22, 66].

#### 4.2.3. Substance Use

Students with consumption habits (coffee, tobacco, cannabis, or other psychoactive substances) revealed more anxiety and depressive symptoms than those who did not consume. Similar associations have been found in other studies. A study with a sample of university students showed that higher levels of anxiety and depression were associated with higher caffeine consumption (*p* < 0.05) [67]. Among the reasons students gave for caffeine consumption were perceived mood enhancement (18%) and stress relief (9%) [68]. A study developed with Brazilian students [69] showed that the problems most reported by those who used drugs were related to depressed mood and difficulties in affective, social, and family relationships. It is possible, in some students, that substances may be used as a maladaptive coping strategy to regulate feelings of hopelessness, depression, or anxiety. In this sense, it is important that when planning strategies to help students reduce their consumption, a prior mental health assessment should be done to address these symptoms by finding alternative coping strategies.

No significant difference was found in alcohol use in this study. Alcohol consumption has a strong social component, and its consumption is composed of a set of positive beliefs and attitudes in which the act of drinking with others facilitates coexistence among people, generating greater disinhibition and interaction among the group [70]. In a longitudinal study of Bangladeshi university students (*n* = 1140), alcohol consumption emerged as a risk factor for depression (*B* = 1.502; *p* = 0.009; *β* = 0.099) and anxiety (*B* = 1.124; *p* = 0.027; *β* = 0.085) [71]. A systematic literature review [72] that included studies from the United States and Canada, Europe, East Asia, South Asia, Saudi Arabia, and Australia, with sample sizes ranging from 60 to 15,396 university students, showed that alcohol as a risk factor for depression and anxiety is not always the case, and when associated with substance abuse, it emerges as a risk factor for suicide. In the Portuguese context, college students socialize at night in bars, accompanying their socialization with alcohol consumption, so it is likely that part of those who consume alcohol are those who socialize more, and this may be the reason there were no significant differences found between those who consume and those who did not consume, considering the phase of life they were in.

#### 4.2.4. Moral or Sexual Harassment

The results of this study show that more than half of the students reported they had suffered from moral and/or sexual harassment. These students reported relatively more anxiety and depressive symptoms. On the one hand, those who are more psychologically vulnerable are more likely to suffer from harassment; conversely, those who suffer from harassment are more likely to experience mental health problems. It is important to take steps to continually assess students' mental health and to monitor harassment by seeking healthier academic environments.

Regarding sex, women most reported having suffered from both types of harassment. A study conducted with North American students [73] showed that 47% of women reported sexual harassment, evidencing increased levels of mental health problems. So, actions that promote gender equality should be promoted. In the same sense, a study developed by Jussen et al. with 579 students found that 67% of those who experienced harassment were female [74]. Regarding moral harassment, most aggressors were family members (26.7%), and for sexual harassment, aggressors were nonteaching staff (14.0%) and love partners (10.5%). An Austrian study [75] showed that students experienced harassment mainly from strangers (79.5%), friends (75.0%), and university staff (68.2%) who exerted power over students, further revealing that most students were female. Furthermore, most of both types of harassment occurred outside the university, so it is important not only to take remedial action within the university but also in the community, given the context in which students live.

In a qualitative study of sexual harassment by Bloom et al. [76], with North American postgraduate students, there was a feeling of lack of information on the subject at the university, a “grey area” in relationships with staff and teachers, leading to a feeling of vulnerability, some impunity on the part of those perpetrating aggression, a lack of confidence in the institution in resolving problems of aggression, and the recognition of the need for cultural and institutional change. There is a need for the increased provision of education and training programs for students and faculty members, optimization of support services (e.g., counselling and advocacy), and further studies that may lead to changes in institutional policies and procedures.

### 4.3. Strengths and Limitations of This Study

Our results should be interpreted according to the context of the study design and limitations. The results were obtained with self-report questionnaires, showing the students' mental health perspective, and our results should not serve as a means of clinical diagnosis of mental health problems. Convenience sampling was used in an environment where most students were female; there was considerable age asymmetry; and most participants were undergraduate or graduate students in their first year of a health science degree. Some of these sample characteristics can lead to potential biases. This was a cross-sectional study that did not consider periods of major or minor stressors that probably influence students' mental health (such as during “exam season”); therefore, longitudinal studies are needed to evaluate the evolution of mental health, and future studies should evaluate the impact of actions to promote the mental health of university students.

The study's strengths include its considerable sample size and the representativeness of students from seven different higher education institutions. The obtained results are relevant and deserve further investigation. Overall, we see our study as contributing to the awareness of the problem of the mental health of university students, already a vulnerable population, one that was experiencing constant socioeconomic changes during COVID-19 and a trending decrease in mental health. This study hopefully also contributes to changing institutional procedures and policies in Portugal, to promote the well-being of university students. Our results provide a snapshot diagnosis of the mental health situation of our particular participants. Our results could help to contribute to building mental health promotion programs for Portuguese university students in a centralized and integrated way. This type of study makes it possible for universities in various countries to work together, contributing to benchmarking methodology.

## 5. Conclusions

We explored the mental health of Portuguese university students in a period when most of the restrictions imposed due to COVID-19 were already lifted. There was a high prevalence of self-reported anxiety and depressive symptoms. We found an association between these symptoms and sex, socioeconomic status, previous mental disorder, substance use, academic performance, return-home frequency, area of study, and moral or sexual harassment. We also observed fewer depressive symptoms in working students and fewer anxiety symptoms in those not in a romantic relationship.

Overall, results possibly indicate that Portuguese university environments are not yet prepared to promote students' mental health, making it difficult to return after the COVID-19 pandemic to school activities in healthy environments that promote well-being. It is necessary and urgent to reflect on pandemic-related changes in academic environments to promote the well-being of students, preventing the onset of mental disorders and disabilities. It seems necessary to invest in mental health literacy actions, as they allow the recognition of signs and symptoms in oneself or in others, the design of adaptation strategies, or even first-aid competencies in mental suffering. There is also a need to review and improve socioeconomic support policies for students, reducing economic imbalances, benefiting mental health, and reducing school dropouts. Finally, the data collected suggest the need for programs that train students to define and practice strategies for dealing with everyday stressors.

Also evident is the need to keep an eye on the mental health status of university students, considering academic periods that may be relatively more stressful and which groups are most vulnerable. Aware of the complexity of the phenomenon, we should all be involved. Researchers, university stakeholders, health services, society in general, policy-making bodies, and the students themselves, ideally in a concerted way, can help to promote the mental health of university students.

The results of this study hopefully contribute to helping university decision-making bodies, politicians, health professionals, and society in general to understand the current mental health of university students. Our findings might also both be compared with data from other countries and contribute to the construction of models that promote the mental health of university students.

## Figures and Tables

**Table 1:** Sociodemographic and academic characteristics (*n* = 3,399).

Variable	Categories	*n*	Valid %
Sex	Female	2300	68.0
	Male	1082	32.0

Loving relationship	No love relationship	1761	51.2
	In a love relationship, without living together	1218	36.1
	In a loving relationship, living together	336	10.0
	Other	60	1.7

Sexual orientation	Heterossexual	2792	83.0
	Bisexual	304	9.0
	Homossexual	132	3.9
	I prefer not to answer	86	2.6
	Other	51	1.5

Nationality	Portuguese	3211	95.0
	Other	168	5.0

Socioeconomic level (students' perceptions)	Low	199	5.9
	Medium	2929	86.5
	High	256	7.6

Relocated from the official residence	Yes	1791	52.7
	No	1606	47.3

If displaced, how often do you go home?	Every weekend	649	36.6
	Two to three times a month	419	23.7
	Once a month	231	13.0
	Only during school breaks/school holidays	473	26.7

With those who live in class time	Parents	1369	40.3
	Study colleagues/friends	1262	37.1
	Other relatives	384	11.3
	Alone	344	10.1
	Boyfriend or girlfriend	268	7.9
	Other	197	5.8

Working student	Yes	518	15

University Type	Public	2923	86.4
	Private	460	13.6

Academic performance (student's perceptions)	Mediocre	253	7.5
	Sufficient	986	29.1
	Good	1703	50.3
	Very good	387	11.4
	Excellent	57	1.7

**Table 2:** Median (ME) and interquartile range (1st and 3rd quartile) of GAD-7 and PHQ-9 scores and *p* value (*p*) from the Mann–Whitney-Wilcoxon or the Kruskal-Wallis tests.

Variable	Categories	GAD-7		PHQ-9	
		ME (IQR)	*p*	ME (IQR)	*p*
Sex	Female	8 (5, 13)	**<0,001**	8 (4, 13)	**<0.001**
	Male	6 (3, 10)		6 (2, 10.75)	

Love relationship	No relation	8 (4, 12)	**0.040**	8 (3, 13)	0.128
	In a relation	8 (5, 12)		7 (3, 12)	

Socioeconomic level (students' perceptions)	Low	8 (6, 14.5)	**0.005**	10 (5, 17)	**0.001**
	Medium	7 (5, 12)		8 (3, 13)	
	High	6 (3, 10)		6 (2, 11)	

Displaced from official residence	No	7 (4, 12)	0.459	7 (3, 13)	0.325
	Yes	7 (5, 12)		8 (3, 13)	

If displaced, frequency going home	Every weekend	7 (4, 11)	**0.015**	7 (3, 11)	**<0.001**
	Two to three times a month	8 (5, 12)		8 (4, 13)	
	Once a month	8 (5, 12)		8 (4, 12)	
	Only on school breaks/holidays	8 (5, 13)		9 (4, 14)	

Previous mental disorder diagnoses	No	7 (4, 11)	**<0.001**	6 (3, 11)	**<0.001**
	Yes	11 (7, 15)		12 (7.75, 17)	

Course year	First	7 (4, 12)	0.610	7 (3, 13)	0.874
	Other year	7 (5, 12)		8 (3, 12)	

Academic performance (student's perceptions)	Mediocre	10 (7, 14)	**<0.01**	12 (7, 18)	**<0.01**
	Sufficient	8 (5, 13)		9 (4, 15)	
	Good	7 (4, 12)		7 (3, 11)	
	Very good/excellent	7 (4, 11)		5 (2, 10)	

Working student	No	7 (5, 12)	0.516	8 (3, 13)	**0.026**
	Yes	7 (4, 12)		7 (3, 12)	

Drink coffee	No	7 (4, 12)	**0.019**	6 (3, 11)	**<0.001**
	Yes	7 (5, 12)		8 (3, 13)	

Smoke tobacco	No	7 (4, 12)	**<0.001**	7 (3, 12)	**<0.001**
	Yes	8 (5, 12)		9 (4, 14)	

Drink alcoholic beverages	No	7 (4, 13)	0.856	8 (3, 14)	0.297
	Yes	7 (5, 12)		8 (3, 12)	

Drink more than 4-5 alcoholic drinks on the same occasion	No	7 (4, 13)	0.860	7 (3, 12)	0.194
	Yes	7 (5, 12)		8 (3, 13)	

Use cannabis	No	7 (4, 12)	**<0.001**	7 (3, 12)	**<0.001**
	Yes	8.5 (5, 13)		10 (5, 15)	

Use other illegal psychoactive substances	No	7 (4, 12)	**0.030**	8 (3, 13)	**0.002**
	Yes	9 (7, 13.25)		10 (6, 16)	

Bold *p* values represent significance at 0.05.

## Data Availability

Due to data protection restrictions in the country and ethics commission rules, as the data is sensitive data, it cannot be made freely available. Partial data that does not jeopardize data protection can be requested from the study coordinator at lmgp@uevora.pt.
