# Correction to “Depression and Anxiety of Portuguese University Students: A Cross‐Sectional Study about Prevalence and Associated Factors”

**DOI:** 10.1155/da/9832346

**Published:** 2026-08-04

P. Amaro, C. Fonseca, A. Afonso, et al., “Depression and Anxiety of Portuguese University Students: A Cross‐Sectional Study about Prevalence and Associated Factors,” *Depression and Anxiety* 2024 (2024): 5528350, https://doi.org/10.1155/2024/5528350.

In the article titled “Depression and Anxiety of Portuguese University Students: A Cross‐Sectional Study about Prevalence and Associated Factors,” there was an error in the “Results” section, under the subsection “Mental Health Data” (page 4). The text originally read:

“Regarding depressive symptoms, participants scored between 0 and 27 on the PHQ‐9 (total scale); the mean was 8 59 ± 6 43, and 38.8% of the participants had moderate or several symptoms (Figure 3).”

This should have read:

“Regarding depressive symptoms, participants scored between 0 and 27 on the PHQ‐9 (total scale); the mean was 8 59 ± 6 43, and 37.5% of the participants had moderate to severe symptoms (Figure 3).”

We apologize for this error.

